# Biopriming of Maize with their endophyte *Aspergillus fumigatus* reinforces their resistance to salinity stress and improves their physiological traits

**DOI:** 10.1186/s12870-024-05871-2

**Published:** 2024-12-30

**Authors:** Marwa A. Yassin, Nelly George, Lamis Shabaan, Yousra Gouda

**Affiliations:** Botany and Microbiology Department, Faculty of Science, Zagazig University, Zagazig, 44519 Egypt

**Keywords:** Maize, *Aspergillus fumigatus*, Biopriming, Salt stress, Gene expression

## Abstract

*Zea mays* L. (Maize) is one of the most crucial world’s crops, for their nutritional values, however, the water scarcity and consequent soil salinization are the major challenges that limit the growth and productivity of this plant, particularly in the semi-arid regions in Egypt. Recently, biopriming has been recognized as one of the most efficient natural-ecofriendly approaches to mitigate the abiotic salt stress on plants. The haploid (128) and triploid (368) seeds of maize were selected as model verities for assessing their resistance to salt stress and mitigating their effect by fungal-biopriming. Overall, the haploid and triploid plants viabilities were drastically affected by salt concentration, at 500 mM of NaCl. At 500 mM NaCl, the fresh weights of the triploid and haploid seedlings were reduced by ~ 5 and 6.1 folds, compared to the controls, ensuring slightly higher salt resistance of the triploid than haploid ones. The pattern of the endophytic fugal isolates was plausibly changed with the salt concentration for both plant types, *Aspergillus fumigatus* isolate was emerged with the higher NaCl concentration (400–500 mM), and their morphological identity was molecularly confirmed and deposited into Genbank with accession # PQ200673. The fungal bioprimed seeds of the haploid and triploid plants were irrigated with 400 mM NaCl. The fungal-bioprimed plants displayed a significant improvement on the shoot density, fibrous roots, root length, shoot length, and leaves numbers and areas of the stressed-plants by ~ 1.7 folds, compared to control, ensures the triggering of different salt resistance machineries in plants upon fungal biopriming. The total antioxidant enzymes activities “catalase, peroxidase, superoxide dismutase” of the salt-stressed bioprimed maize plants were increased by ~ 4.7–5.3%, compared to control, confirming the mitigating effect of the salinity stress on plants upon fungal biopriming. The chlorophyll and carotenoids contents were significantly increased of the salt stressed maize upon biopriming with *A. fumigatus*. The expression of the *sod*, *apx*2, *nhx1*1, *hkt*1, *H* + *-PPase*, *nced* of the plant salt stressed was strongly increased in response to *A. fumigatus* biopriming, normalized to β-actin gene. The expression of *apx*2 was dramatically increased by about 30 and 43 folds, in response to fungal biopriming. The *nhx*1 was significantly up-regulated by 18.9 fold in response to fungal biopriming, compared to control.

## Introduction

Maize (*Zea mays* L.) is one of the most important world’s crops, due to their high nutritional value, high levels of starch, valuable proteins, oils, vitamins and essential elements [1]. Maize has been considered as a valuable source of biofuels and feed [2], in which 100 g of Maize provides 365 kilocalories [3], thus, providing half of the world's calorie consumption [4]. Maize, as C4-plants, has a high growth rate, with a relatively short lifespan, however, with moderate sensitivity to salt stress [5, 6]. Salinity is one of the severe environmental abiotic stresses that restricts the productivity and sustainability of agricultural crops in the arid and semiarid regions [7, 8]. In addition, the exaggerating effects of global warming, inappropriate irrigation methods, excessive use of fertilizers and pesticides, cause a marked worsening of soil salinization around the world [2, 3]. Additionally, the excessive accumulation of salt in the plant’s cells produces a reactive oxygen species (ROS), especially hydrogen peroxide, hydroxyl radicals, and superoxide anions that subsequently inhibits all the cellular biological process [9, 10]. For alleviating the salt toxicity and surviving, the plants can resist the salt stress by developing various mechanisms like extrusion of salts, biosynthesis of osmolytes, and triggering of the ROS scavenging systems [11], generating several osmolytes such as proline, sorbitol and glycine betaine to minimize the plant osmotic potential by maintaining water uptake level and cell turgor [12].

Soil salinity is considered as a challenge in the agricultural field in Egypt, in which 33% of the cultivated lands which occupies 3% from the whole land area in Egypt suffers from the severe salinity [13]. Primary and secondary soil salinization, arising from salt stress, has a major impact on the decline of plant productivity, especially in the arid and semi-arid zones, due to the scarcity of rain, high evaporation rate and deterioration of water quality that leads to soluble salts accumulation in soil surface [14]. The salt stress diminished the plant growth by osmotic induction of water stress, elevating the sodium and chloride concentrations, causing unbalanced nutrition, rising up of Na, K, and PO_4_ absorption, increasing the ROS production with a deleterious effect to the macromolecules [15]. Salinity induces stomatal closure, leads to reduction in CO_2_ fixation and increasing ROS (reactive oxygen species) generation [16, 17], causing oxidative constituents cellular damage, lipid peroxidation, DNA damage, protein degradation and enzyme deactivation [18]. It has been reported that salt stress has visual symptoms such as appearing of wilting symptoms, yellowish of leaves and stunting in growth, chlorosis, burning in leaf tip and scorching of older leaves [19]. Salinity decreases the stomatal conductance, diminishes the net photosynthesis, alters the chloroplast ultrastructure, reduces the carboxylation reactions, elevating the soluble sugars level in tissues [20].

Several approaches have been implemented to fortify the metabolic and physiological potency of plants to tolerate the salt stress [21]. Enhancing the maize's resistance to salt may be possible through the integration of breeding process, application of exogenous hormone, and osmo-protectants application for seeds or cultivars, ion homeostasis and cellular-based stress signaling in a model of function paradigm for the entire plant [22]. Recently, microbial biopriming is one of the most common practical approaches to acquire the plant biological defense against abiotic stress by developing a specific signal for plant resistance to the different biotic and abiotic stresses [23]. Biopriming mediates the molecular and metabolic reprogramming of the plant cells to impart the stress tolerance, improves the plant health, and enhances crop productivity [24]. It has been reported that biopriming technique promotes vigor indices, seed viability, plant growth, germination and subsequently protection against disease [23, 25, 26].

Endophytic fungi are mainly involved in the tolerance of plants to the environmental stresses, including salt stress, herbivores, drought, pathogens, and paucity of nutrients [27, 28]. The symbiotic plant-endophyte relationship has been relied on mitigating the environmental stresses on plants, by secreting anti-stress phytohormones by the fungal endophytes that increases the root hair growth, root area, promoting the activity of ACC (1-aminocyclopropane-1-carboxylate deaminase) that involved in reduction of ethylene level, production of antioxidative defense osmolytes [26, 29–32]. However, the biological identity of the fungal endophytes of *Zea mays* and their relation to the resistance of the plant to the abiotic stress (particularly salinity), has received less attention. So, the objective of this study was to explore the fungal endophytes of the plant in response to salinity stress, to fortify the plant resistance to salinity, to mitigate the salt stress via the fungal biopriming approaches.

## Materials and methods

### Plant material and growing conditions

Grains of triploid maize (368) and monoploid maize (128) were obtained from the Seeds Facility Unit, Agriculture Research Center, Egypt. The grains were washed three successive times with sterilized distilled water, surface sterilized by with 70% ethanol, and commercial Clorox bleach solution [33]. The sterilized grains were presoaked in sterilized distilled water for 2 h, then allowed to grow in plastic pots (30 cm deep × 25 cm wide × 40 cm long) of 1 kg of sandy-clay soil (sand particles 80.5%, silt 7.1%, clay 8.1%, organic carbon 4.10%, and total nitrogen 0.83%), under the standard light and dark conditions with normal tap water for irrigation. The pots were incubated at an average day-night temperature 34 ± °C to 22 ± 3 °C. After 16 days of plant growth, the seedlings were irrigated with water amended with various salt concentrations (100, 200, 300, 400, and 500 mM) with regular irrigation till 35 days. The morphological features such as plant viability, shoot length, root length, chlorophyll contents, were assessed [21, 24].

### Isolation of the endophytic *fungi* and selection of the most potent isolate

The endophytic fungi from both plant types of Maize grown at different salt concentration were isolated as described by [34], with slight modifications. Briefly, leaves of the plant grown at different salt concentration were gathered, washed by sterile distilled water, and surface sterilized by 70% ethanol for 1 min, and 2.5% sodium hypochlorite for 1 min, then the leaves were segmented into 5 mm long using sharp blade. The leaves segments were dried with sterile filter paper, subsequently, the plant sections were placed on the surface of potato dextrose agar (PDA) (200 g potato extract, 20 g glucose, and 20 g agar per liter), with 1 μg/ml ampicillin, then the cultures were incubated at 30 °C for 15 days. The developed fungal colonies were collected, purified, stored as slope cultures [33, 34]. The purified endophytic fungal isolates were stored as slope cultures at 4 °C. The isolated fungi were identified based on their morphological features according to the universal keys [35, 36].

### Molecular identification of the isolated endophytic *fungi*

The identity of potent fungal isolates was molecularly confirmed based on the sequence of their internal transcribed spacer (ITS), flanking the 5.8 S region (ITS1-5.8S-ITS2) [24]. The fungal genomic DNA (gDNA) was extracted by cetyltrimethyl-ammonium bromide (CTAB) [34, 37]. The fungal gDNA was used as a PCR template with the primers ITS4 5′-GAAGTAAAAGTCGTAACAAGG-3′ and ITS5 5′-TCCT-CCGCTTATTGATATGC-3′. The PCR reaction contains 2 μl of gDNA, primers (10 pmol), and 10 μl of 2 × PCR master mixture (Cat. # 25,027), and completed to 20 μl with sterile water. The PCR was programed into initial denaturation 95 °C for 2 min, followed by 35 cycles of denaturation at 95 °C for 30 s, annealing at 55 °C for 30 s, extension at 72 °C for 1 min, and final extension at 72 °C for 5 min. The PCR products were analyzed by 1.5% agarose gel in 1 × TBE buffer, compared to DNA ladder (Cat. #PG010-55DI). The amplicons were sequenced by the Applied Biosystems Sequencer, and the retrieved sequences were non-redundantly BLAST searched on the NCBI. The phylogenetic relatedness of the fungal sequence was analyzed by Clustal W muscle algorithm at 1000 bootstrap replications, with the neighbor-joining tool by MEGA X [38, 39].

### Biopriming of maize grains with the potent fungal isolates

The selected fungal isolates from both types of Maize were grown in potato dextrose broth (PDB) under static conditions at 30 °C for 10 days. After incubation, the fungal cultures were filtered, the fungal biomass were washed with sterile saline solution. The grains of Maize were bioprimed with the selected fungal isolates as described by [40], with slight modification. Breifly, the experiment was designed as follows; Group 1, controls of maize grains of types 368 and 128, without salt or fungal treatment. Group 2, Maize grains of types 368 and 128 grown on sandy-clay soil irrigated with water of 400 mM NaCl. Group 3, Maize grains of types 368 and 128 bioprimed with the powdered mycelia of the tested fungus, irrigated with water of 400 mM NaCl. Group 4, Maize grains bioprimed with the fungus irrigated with water with zero salt. Fifteen treated seeds were sown per pot of 3 kg of sandy-clay soil, allowed to germinate. The sandy-clay soil composed of 80.5% sand particles, 7.1% silt, 8.1% clay, 4.10% organic carbon, 0.83% total nitrogen. The pots were incubated at an average day-night temperature 34 ± °C to 22 ± 3 °C. After 16 days of growth and irrigation with tap water, the seedlings were irrigated with 500 ml water of 400 mM NaCl, every five days. The samples of the plant leaves were collected on the 30th day to assess the various morphological, biochemical and molecular parameters, as follows;

### Morphological features

The morphological features of the plant include shoot length, root length, shoot fresh weight, shoot dry weight, number of leaves, number of fibrous roots, leaf width, leaf length, shoot density and leaf area [21, 33]. A precise description of the presence and absence of different morphological traits for each species before and after inoculation with fungus was reported. The average value of each quantitative character's standard deviation was computed, and the state of the qualitative and present character was recorded.

### Antioxidant enzymes assay

The enzymatic antioxidant activities of the experimented types of maize were assessed [41]. Ten grams of the plant leaves were collected, washed, and pulverized in liquid nitrogen into fine powder, then dispensed in Tris–HCl buffer contains 1 mM EDTA and 1 mM DTT, centrifuged at 5000 rpm for 10 min, and the supernatant was used as source of crude enzyme [33, 34]. The activities of selected antioxidant enzymes were determined.

The activity of catalase (CAT) was estimated by Biodiagnostic Kit (CA.#25–17), according to [42] and [43] Briefly, 0.05 ml of the crude enzyme source, 0.5 ml of phosphate buffer (pH 7) and 0.1 ml of chromogen-inhibitor were mixed and incubated for 1 min at 25 °C, then 0.5 ml H_2_O_2_ were added to the mixture, incubated for 10 min at 37 °C. The decrease in absorbance was recorded at 510 nm, the absorbance of the developed chromophore was inversely proportional to the amount of catalase in the sample.

The activity of glutathione peroxidase (POD) was estimated by the Biodiagnostic Kit (GP 25–24) [44], that based on the decrease in NADPH absorbance during the oxidation of NADPH to NADP^+^ [45]. Briefly, the reaction contains crude enzyme (0.01 ml), 2 mL potassium phosphate buffer (50 mM, pH 7) of 5 mM EDTA and 1 mM 2-mercaptoethanol, 0.1 ml of the NADPH, the reaction was incubated at 25 °C. The decrease in absorbance of NADPH was recorded at λ_340_ nm.

The activity of superoxide dismutase (SOD) was measured by the Biodiagnostic Kit (Cat. #25–21) [46] with slight modifications. Briefly, the reaction mixture contains the crude plant extract, 2 ml of 100 mM potassium phosphate buffer of pH 7.5 and 2 mM EDTA, 1 ml of nitro blue tetrazolium (NBT). The reaction was incubated was for 5 min at 25 °C, and the increase on the absorbance was measured at λ_560_ nm.

### Chlorophylls and carotenoids content

The chlorophyll and carotenoid contents of the experimented plants in response to stress and fungal biopriming were measured [47]. For extraction of chlorophylls (a and b), 100 mg fresh weight of leaves were homogenized in 5 ml DMF (N,N-dimethylformamide), then the samples were centrifuged at 12,000 rpm, for 10 min at 4 °C. The supernatants were collected, the pigments were determined by measuring the absorbance at 663.8 nm for chlorophyll a, and 646.8 nm for chlorophyll b using UV–visible. The chlorophyll a, b concentrations and total carotene (µg/ml) was determined based on the fresh weight (mg/g), according to the following equations. Chlorophyll a = 12.00 (A663.8 nm)−3.11 (A646.8 nm). Chlorophyll b = 20.78 (A646.8 nm)−4.88 (A663.8 nm). The total chlorophyll contents (mg/mL) was the amount of Chlorophyll a + Chlorophyll b. Carotenoids = 1000 (A470nm)-[0.89 (Chl a)−52.02 (Chl b)]/245.

### RT-qPCR molecular expression analysis

#### RNA extraction and cDNA synthesis

The plant leaves from the selected treatments in response to salinity and biopriming with the target fungus, were collected and their total RNA was extracted using the IQeasyTM plus Plant Mini Kit (Cat#. 17,491, iNtRON Biotech. Korea). The plant leaves (0.1 g) were pulverized into fine powder in liquid nitrogen, then the powder was transferred into 2 ml Eppe tubes, and TRIzol (1 ml) was added to cells, homogenized by pipetting for 10 times. Chloroform (200 µl) was added to the homogenate, mixed well by pipetting, vortex vigorously for 5 min, then incubated in ice for 15 min, then the homogenate was centrifuged at 10,000 rpm for 15 min at 4 °C. The aqueous upper phase was transferred into a new tube, and the total RNA was precipitated by 500 µl isopropanol, incubated in ice for 10 min, centrifuged at maximum speed for 15 min at 4 °C, and the supernatant was decanted, and the RNA pellets were washed by 1 ml 70% Ethanol, and dissolved in 25 µl of RNase-free water and stored at −80 °C. The purity of the extracted RNA was checked by the nanodrop spectrophotometer (Thermo Scientific NanoDropTM 1000), regarding to the absorbance ratios (A260/280) and (A260/230). The equivalent total RNA concentration from each sample was adjusted to 10 ng/µl, and the cDNA was synthesized according to ABT H-minus cDNA synthesis Kit manufacturer’s instructions using 5X first strand buffer, 0.5 µl of H minus MMLV (200 U/µl), and 2 µl dNTPs mixture (10 mM), RNA template. The reaction was incubated at 42 °C for 60 min, and stopped by heating for 5 min at 70 °C.

#### RT-qPCR analysis

The expression of the crucial genes of the plant resistance to abiotic stress “salinity” namely; sod4, apx2, NHX1, HKT1, H^+^-PPase, and NCED was assessed by the RT-qPCR, compared to β-actin genes as house-keeping genes. The primers sequences were listed in Table 1. The RT-qPCR reaction contains cDNA, primers, and SYBER Green Mastermix (Grisp, Portugal), according the manufacturer’s instructions (Cat. #0.17491, iNtRON Biotech. Korea). The real-time PCR machine was programed to initial denaturation at 95 °C for 3 min, followed by 40 cycles of 95 °C for 10 s, 55 °C for 15 s, 72 °C for 20 s. Melting curve analyses were performed at 72–95 °C. Triplicates of each sample were conducted. The results were normalized to the house-keeping *β-Actin* gene, as endogenous control, and the expression folds of the target genes were calculated using the Rotor-Gene software 1.7.94, based on 2^−ΔΔct^ formula.

**Table 1:** Primer sequences of SOD, APX, NCED, H + -PPase, HKT1 and NHX1 for the RT-qPCR analysis

	**Gene**	**Primer sequence (5′–3′)**
1	β-actin	F: TCGCTGACCGTATGAGCAAAG R: TGTGAACGATTCCTGGACCTG
2	*sod4*	F: TGGAGCACCAGAAGATGA R: CTCGTGTCC ACCCTTTCC
3	*apx2*	F: TGAGCGACCAGGACATTG R: GAGGGCTTTGTCA CTTGGT
4	*nced*	F: CCGCCGACTCCATCTTCAA R: TTCACCATCCCGACCTCCA
5	*H*^+^*-ppase*	F: GGTATTCAGTGGTGTGCTAT R: GGTGGTCCTTCGTCCTTA
*6*	*HKT1*	F: TCTTCATCGTCGTCATCTG R: CCTTCCACACTCCACTTG
7	NHX1	F: ACTTGTTCTTCACCAGCACCATACT R: ATTCCACTCAGGTCCAACAGCATT

#### Statistical analysis

All the experiments were conducted in biological triplicates. Statistical analyses were performed with Costat software program by One Way ANOVA, and Tukey HSD to determine the significant differences among the un-primed maize and fungal-primed ones at *p* < 0.05.

#### Fungal deposition

The most potent fungal isolate, *Aspergillus fumigatus* MY, was deposited into the Genbank with accession number PQ200673.

## Results

### Growth and morphological traits of the haploid and triploid types of maize

**Fig. 1:**
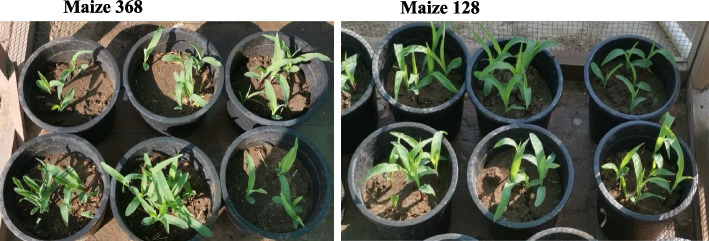
Growth of the haploid maize (128) and triploid maize (368) on sandy-clay soil in greenhouse under standard conditions after 16 days

The haploid (SC-128) and triploid (TWC-368) seeds of maize were selected as model verities for assessing their resistance to salt stress. The plant seeds were grown on standard sandy-clay soil in pots, in greenhouse for 40 days, under standard growth conditions. The overall growth of the seedlings of the two types of maize seeds was observed and photographed (Fig. 1). The obvious morphological differences on the seedlings of the haploid (single cross) and triploid (three-way cross) types of maize were observed. The cultivars SC-128W had a leaf area index of 754.6, while, the cultivars TWC-368 has three-way cross had the highest index at 804.0. Furthermore, TWC-368 had a greater seedling rate (33.33 kg/ha) than SC-128W (28.57 kg/ha) [48]. The height of the TWC-368 and Sc-128 under regular watering and water stress were 248.12, and 240.00 cm, respectively [49].

**Fig. 2:**
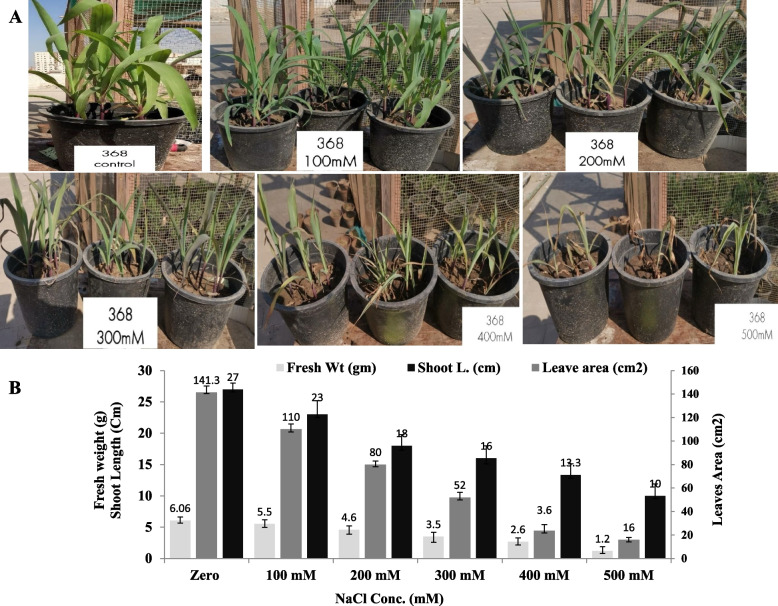
Growth of the triploid Maize (368) seedlings at different salt concentrations. **A** Morphological appearance of triploid maize at different NaCl concentrations. **B** Fresh weight, Shoot length and Leaves area of the triploid plant irrigated with different salt concentrations

**Fig. 3:**
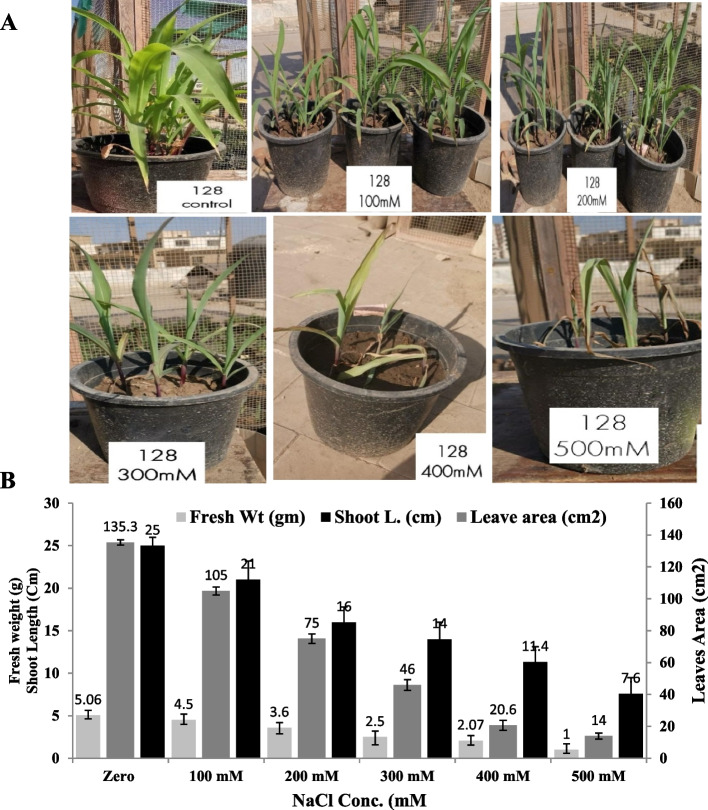
Growth of the Haploid Maize (128) seedlings at different salt concentrations. **A** Morphological appearance of triploid maize at different NaCl concentrations. **B** Fresh weight, Shoot length and Leaves area of the haploid plant irrigated with different salt concentrations

The physiological resistance of the two types of maize was assessed in response to different salt concentrations. The seeds were planted into sandy-clay soil and after two weeks of growth, the plants were irrigated with water amended with different salt concentration, as described in Materials and Methods. The growth parameters of the triploid maize (TWC-368) and haploid maize (Sc-128) seedlings at different salt concentrations, such as fresh weight, dry weight, leaf length. leaves width, diameter of stem, number of fibrous roots, root length, shoot length, and number of plant leaves were recorded in the Tables 2 and 3. The least significant difference of the various plant physiological paramters in response to salt concentration was estimated were assessed. From the physiological parameters of both types of plants (Fig. 2), the overall morphological appearance of the two plants was drastically affected by the higher salt concentration. The different physiological parameters mainly fresh weight, shoot length and leave areas were measured for the haploid maize and triploid maize (Figs. 2, 3). The fresh weight, shoot length and leaves area were gradually reduced with the higher salt concentration, till maximum reduction at 500 mM NaCl. At 500 mM NaCl, the fresh weight of the triploid seedling was reduced into 1.2 g compared to zero salt (plant fresh weight 6.06 g), i.e. by about 5 folds reduction compared to control. At 400 mM NaCl, the shoot length of the triploid maize was reduced from 141.3 cm (zero salt) into 23.6 cm, i.e. by about 5.9 reduction folds. Similarly, the leaves area was drastically reduced by about 2.7 folds at 500 mM NaCl compared to zero salt concentration. As well as, from the physiological parameters of the haploid plant maize (SC-128), the fresh weight, shoot length and leaves area of the plant seedlings were drastically affected by the higher salt concentration. At 500 mM NaCl, the fresh weight, shoot length and leaves area were reduced by about 5.2, 9.6 and 3.4 folds, respectively, compared to zero salt plant (Fig. 3). From the comparative results, the triploid maize plants had a slight resistance to the salt stress than the haploid one, as revealed from the fresh weight, shoot length and leaves area of the plants.

**Table 2:** Growth parameters of the triploid maize (TWC-368) seedlings at different salt concentrations. Fresh weight, dry weight, leaf length. Leaves width, diameter of stem, number of fibrous roots, root length, shoot length, and number of leaves of the plant at different salt concentrations

NaCl conc. (mM)	Fresh W. (g)	Dry W (g)	Leaf L (cm)	Leaf width (cm)	Diameter of stem (cm)	No. of fibrous roots	Root L (cm)	Shoot L (cm)	No. of leaves
100	**4.327 ± 0.43**^**a**^	**1.0186 ± 0.15**^**a**^	**33 ± 3 a**	**1.68 ± 0.29**^**a**^	**1.9 ± 0.1**^**a**^	**6.67 ± 2.08**^**ab**^	**10.16 ± 2.75**^**a**^	**11.166 ± 1.60**^**a**^	**5.33 ± 0.57**^**a**^
200	**3.296 ± 0.484**^**ab**^	**0.769 ± 0.050**^**abc**^	**28.67 ± 2.020**^**ab**^	**1.68 ± 0.29**^**a**^	**1.86 ± 0.32**^**a**^	**7.33 ± 1.52**^**ab**^	**11.67 ± 4.53**^**a**^	**8.33 ± 1.040**^**ab**^	**4.33 ± 0.577**^**ab**^
300	**3.54 ± 0.183**^**ab**^	**0.750 ± 0.067**^**abc**^	**23.33 ± 4.163**^**bc**^	**1.55 ± 0.13**^**a**^	**1.8 ± 0.26**^**a**^	**7 ± 3**^**ab**^	**6.5 ± 1.32**^**a**^	**8 ± 1**^**ab**^	**4 ± 0**^**ab**^
400	**2.29 ± 0.723**^**bc**^	**0.55 ± 0.1380 **^**bc**^	**18 ± 0.5**^**c**^	**1.72 ± 0.165**^**a**^	**1.8 ± 0.36**^**a**^	**7 ± 2**^**ab**^	**10 ± 2**^**a**^	**8.67 ± 2.36**^**ab**^	**4.33 ± 0.577**^**b**^
500	**1.61 ± 0.509**^**c**^	**0.399 ± 0.135**^**c**^	**20 ± 3.605**^**c**^	**0.99 ± 0.383**^**a**^	**1.4 ± 0.35**^**a**^	**4.33 ± 0.577**^**b**^	**9.5 ± 1.32**^**a**^	**6.87 ± 1.03**^**b**^	**4 ± 0**^**b**^
Control	**3.78 ± 0.823**^**ab**^	**0.92 ± 0.28**^**ab**^	**28.67 ± 1.25**^**ab**^	**1.796 ± 0.45**^**a**^	**1.83 ± 0.25**^**a**^	**11.33 ± 3.055**^**a**^	**12.57 ± 2.37**^**a**^	**9 ± 0.87**^**ab**^	**5 ± 0**^**ab**^
LSD _0.05_	**1.004**	**0.277**	**4.607**	**0.53**	**0.51**	**3.93**	**4.66**	**2.52**	**0.726**
*p*	.0007***	.0039**	.0001***	**.0596 ns**	**.3562 ns**	.0481*	**.1672 ns**	**.0535 ns**	.0079**

Values given are **mean ± SD**

The mean values followed by different letters a, b, c with in the same column are significantly different (ONE Way ANOVA, Tukeyʾs test, *p* ≤ 0.05)

***n.s*** non-significant, ***LSD*** the least significant difference

*Significant difference

**means highly significant

**Table 3:** Growth parameters of the haploid maize (SC-128) seedlings at different salt concentrations. Fresh weight, dry weight, leaf Length. Leaves width, diameter of stem, number of fibrous roots, root length, shoot length, and number of leaves of the plant at different salt concentrations

**NaCl Conc. (mM)**	**Fresh W. (g)**	**Dry W (g)**	**Leaf L (cm)**	**Leaf Width (cm)**	**Diameter of stem (cm)**	**No. of Fibrous R**	**Root L (cm)**	**Shoot L (cm)**	**No. of leaves**
100	**4.88 ± 0.15**^**a**^	**1.56 ± 0.36**^**a**^	**31.67 ± 4.25**^**a**^	**2.096 ± 0.430**^**a**^	**2.033 ± 0.057**^**ab**^	**6.66 ± 0.58**^**ab**^	**16.5 ± 5.22**^**a**^	**7.83 ± 1.26**^**a**^	**5.33 ± 0.57**^**a**^
200	**4.347 ± 0.41**^**a**^	**1.55 ± 0.57**^**a**^	**27 ± 3.28**^**ab**^	**2.04 ± 0.33**^**a**^	**2.7 ± 0.7**^**a**^	**7 ± 1.73 ab**	**17.5 ± 2.179**^**a**^	**6.866 ± 1.026**^**a**^	**5.67 ± 0.58**^**a**^
300	**2.81 ± 1.22**^**ab**^	**0.70 ± 0.30**^**ab**^	**20.77 ± 3.219**^**bc**^	**1.56 ± 0.3**^**a**^	**1.93 ± 0.21**^**ab**^	**5.66 ± 1.154**^**ab**^	**13.43 ± 6.501**^**ab**^	**6.533 ± 0.808**^**a**^	**4.66 ± 0.58a**
400	**1.21 ± 0.07**^**b**^	**0.23 ± 0.060**^**b**^	**13.33 ± 2.51**^**c**^	**1.14 ± 0.612**^**a**^	**1.53 ± 0.378**^**b**^	**6 ± 1**^**ab**^	**6.167 ± 1.607**^**b**^	**5.33 ± 1.1547**^**a**^	**4.33 ± 0.58**^**a**^
500	**1.34 ± 0.26**^**b**^	**0.23 ± 0.097**^**b**^	**16.5 ± 3.27**^**c**^	**1.03 ± 0.412**^**a**^	**1.23 ± 0.15**^**b**^	**4.33 ± 0.58**^**b**^	**5.17 ± 0.76**^**b**^	**5 ± 0.5**^**a**^	**4 ± 1**^**a**^
Control	**4.047 ± 1.63**^**a**^	**1.091 ± 0.52**^**ab**^	**26.6 ± 2.28**^**ab**^	**1.32 ± 0.15**^**a**^	**1.93 ± 0.115**^**ab**^	**10.66 ± 3.78**^**a**^	**11.5 ± 0.86**^**ab**^	**8.33 ± 2.362**^**a**^	**6 ± 1**^**a**^
LSD _0.05_	**1.5228**	**0.663**	**5.696**	**0.7087**	**0.614**	**3.274**	**6.421**	**2.348**	**1.325**
P	.0006***	.0017**	.0001***	.0250*	.0045**	.0216*	.0048**	**.0546 ns**	.0396 *

Values given are **mean ± SD**

The mean values followed by different letters a, b, c with in the same column are significantly different (ONE Way ANOVA, Tukeyʾs test, *p* ≤ 0.05)

***n.s*** non-significant, ***LSD*** the least significant difference

*Significant difference

**means highly significant

### Endophytic fungal pattern of the triploid and haploid maize in response to the salt stress

The fungal endophytic profile was assessed after 30 days of plant growth, by platting the plant surface sterilized-sectioned leaves on potato dextrose agar as described in Materials and Methods. The emerged fungal endophytes were counted, collected and purified by subculturing on the same media. Twelve fungal isolates were recovered from the plant’s seedlings at different salt concentration as reported in Table 4. An obvious fluctuation on the frequency and identity of the recovered fungal isolates was observed from the zero-salt control with the salt-stressed maize plants. The control plants were the richest one with the fungal endophyte, with an obvious decreasing to the load of endogenous fungi with the higher salt concentration. From the morphological inspection of the recovered fungal endophytes, the frequency of some fungal isolates was observed to be increased with the salt concentration (400 mM, 500 mM), compared zero salt controls of both types of maize. Among the most conspicuous isolates, that obviously persistent with higher frequency at 400 and 500 mM NaCl, was morphologically identified as *Aspergillus fumigatus,* according the microscopical and macroscopical features [36, 50]. The fungal isolate on PDA medium has an oval-globose conidial heads, conidial mass of conidiophores and mycelia of blue-green shades, uniserriate strigma, lack of sclerotia, conidial masses compactly columnar, these morphological features were closely identical to *Aspergillus fumigatus*.

**Table 4:** The frequence of the edophytic fungi of haploid and triploid maize under salt stress

		**Haploid Maize (SC-128)**						**Triploid (TWC-368)**					
		**Salt concentration (mM)**						**Salt concentration (mM)**					
		Zero	100	200	300	400	500	Zero	100	200	300	400	500
**1**	***Aspergillus niger***	** + + + **	** + + + **	** + + + **	** + + + **	** + + + **	** + + + **	** + + + **	** + + + **	** + + + **	** + + + **	** + + + **	** + + + **
**2**	***Penicillum notatum***	** + + **	** + + **	** + + **	** + + **	** + + **	** + + **	** + + **	** + + **	** + + **	** + + + **	** + + + **	** + + + **
**3**	***Penicillim citrinum***	** + + **	** + + **	** + **	**-**	**-**	**-**	** + **	** + **	** + **	** + **	** + **	** + **
**4**	***Trichoderma* sp**	** + + **	** + + **	** + + **	** + + **	** + + **	** + + **	** + **	** + **	** + **	** + **	** + **	** + **
**5**	***Mucor pusilus***	** + + **	** + + **	** + **	** + **	** + **	** + **	** + + **	** + + **	** + **	** + **	** + **	** + **
**6**	***A. flavus***	** + + **	** + + **	** + + **	** + + **	** + + **	** + + **	** + + **	** + + **	** + + **	** + **	** + **	**-**
**7**	***A. fumigtaus***	** + **	** + **	** + + **	** + + + **	** + + + **	** + + + **	** + **	** + **	** + + **	** + + + **	** + + + **	** + + + **
**8**	***A. tamarii***	** + + **	** + + **	** + + **	** + **	** + **	** + **	** + **	** + + **	** + + **	** + + **	** + + **	** + + **
**9**	***A. ochraceus***	** + + **	** + + **	** + + **	** + **	** + **	** + **	** + + **	** + + **	** + + **	** + + **	** + + **	** + + **
**10**	***A. flavipes***	** + + + **	** + + **	** + + **	** + + **	** + + **	** + **	** + + **	** + + **	** + **	** + **	** + **	** + **
**11**	***A. terreus***	** + + + **	** + + **	** + + **	** + + **	** + + **	** + **	** + + **	** + + **	** + + **	** + + **	** + + **	** + + **
**12**	***A. oryzae***	** + + + **	** + + **	** + + **	** + + **	** + + **	** + **	** + + **	** + + **	** + + **	** + **	**-**	**-**

** + + +Highly frequent**

** + +Moderate frequent**

** +Less frequent**

**- Absent**

**Fig. 4:**
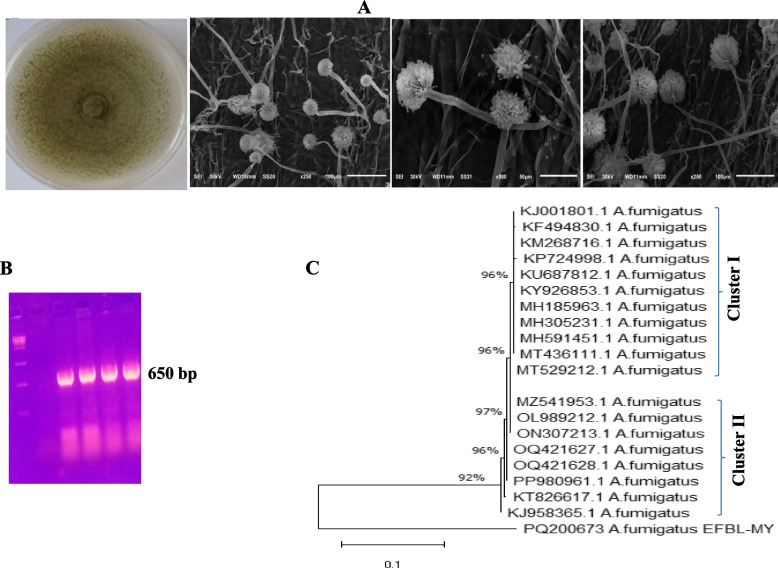
Identification of the most potent endophytic fungal isolate correlated with the maize resistance. **A** The plate culture and conidial heads of A. fumigatus at different microscopic magnifications. **B** PCR amplicon of the ITS region. **C** The molecular phylogenetic relatedness of A. fumigatus based on the sequence of its ITS region by the Maximum Likelihood method

The morphological identity of the potent isolate was confirmed based on their ITS sequence, using fungal genomic DNA as a PCR template. The amplicon of the ITS regions was about 700 bp (Fig. 4). The amplicon was sequenced, and non-redundantly BLAST searched on the NCBI database, gave 100% similarity with the database deposited ITS sequences of *A. fumigatus* with 100% query coverage and zero E-value. The sequence of the ITS region of *A. fumigatus* EFBL-MY was deposited to the Genbank with accession # PQ200673. From the alignment profile and phylogenetic analysis of ITS sequences, the ITS sequences were grouped into two clusters I and II (Fig. 4). The ITS sequence of *A. fumigatus* EFBL-MY was identified as a distinct out-group with ~ 92% similarity to *A. fumigatus* with accession # KJ958365.1, KT826617.1, PP980961.1, OQ421628.1, OQ421627.1, ON307213.1, MZ541953.1, MT529212.1, MT436111.1, MH5914151.1 MH305231.1, MH185963.1, KY926853.1 and KU687812.1, with Zero E value and 99% query coverage. Thus, from the morphological description and molecular fingerprint, the current isolates were identified as *A. fumigatus*.

### Biopriming of the salt stressed maize with* Aspergillus fumigatus*

**Fig. 5:**
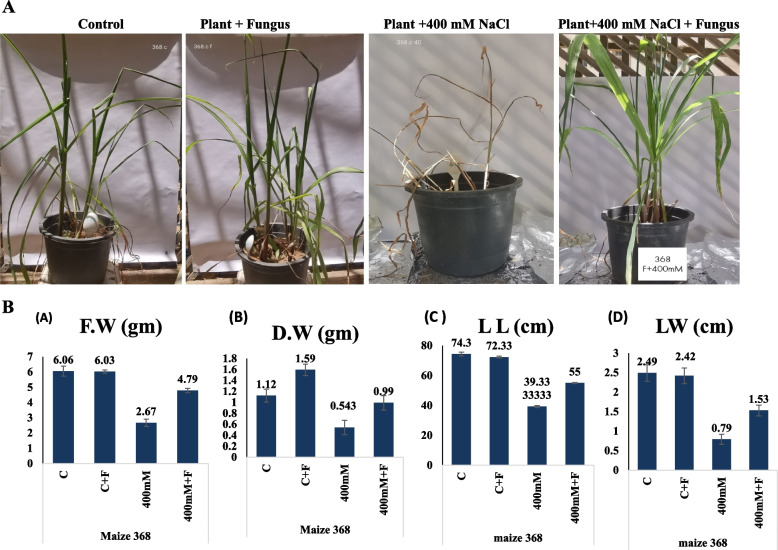
Morphological and physiological responses of the triploid Maize (368) stressed with 400 mM NaCl and bioprimed with A. fumigatus. **A** Visual growth of the salt stressed triploid Maize (368) bioprimed with A. fumigatus. **B** Total Fresh weight (F.W), Dry Weight (D.W), Leave length (L.L) and Leave weight (L.W) of the seedlings after 30 days of growth

**Fig. 6:**
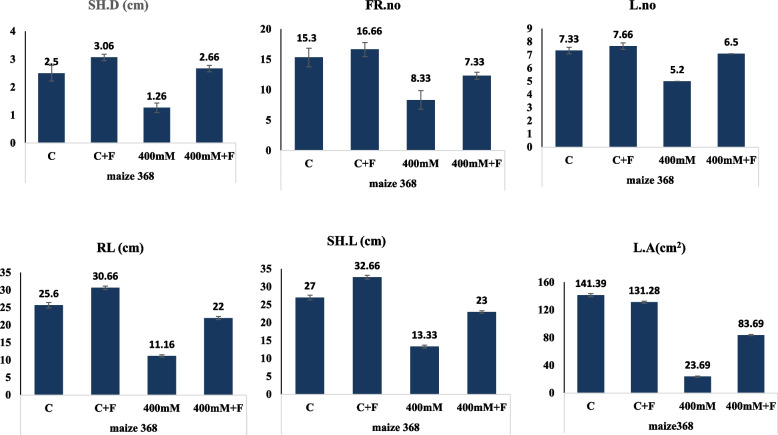
Physiological parameters of the salt stressed triploid plant bioprimed with A. fumigatus. After 30 days of plant incubation, the shoot density (S.D), fibrous roots number (FR, leaves number (L), Root length (RL), shoot length (SL), and leaves area of the triploid maize plants, were assessed

The haploid and triploid maize seeds bioprimed with *A. fumigatus,* were grown under salt stress at 400 mM, in order to assess the effect of fungal biopriming on mitigating the abiotic stress effect of salt. The plant seeds were bioprimed with the *A. fumigatus*, and the seed were planted into sterile sandy-clay soil, as described in Materials and Methods. The morphological growth, viability and various physiological parameters of the experimented plants in response to salt stress and fungal biopriming were assessed (Fig. 5). From the morphological observations, the viability of the non-fungal bioprimed plant seedlings were strongly affected with the NaCl salt at 400 mM, however, the fungal-bioprimed seedlings had an obvious viability as revealed from the different physiological parameters. The fresh weight, dry weight, leaf length, and leaf weight of the triploid maize seedlings bioprimed with *A. fumigatus* were significantly improved, compared to the non-bioprimed plants. The different growth and physiological parameters of the salt stressed triploid plant maize bioprimed with *A. fumigatus* were summarized in Table 5. Interestingly, the viability and physiological parameters of the plants bioprimed with *A. fumigatus* was relatively similar to the non-bioprimed plants, ensuring the lack of toxicity, negative interactions between fungal and plants. The fresh weight and dry weight of the bioprimed plant with the fungus was significantly improved by ~ 1.3 folds, with *p*-value ≤ 0.05, compared to zero salt treated plants. As well as, the leaves length and areas of the triploid plants at 400 mM NaCl, were improved by ~ 1.4 folds upon fungal biopriming, compared to the zero salt. The plant seedlings fresh weight, dry weight, leaves length and widths of the triploid plants were reduced to 50% at 400 mM NaCl, compared to the zero salt-treated plants. As well as, the shoot density, fibrous roots, leaves number, root length, shoot length, and leaves areas of the plants under salt stress, were significantly improved upon biopriming with *A. fumigatus* (Fig. 6). Compared to the salt treated plants (400 mM NaCl), the shoot density, number of fibrous roots, leaves number and root length of triploid maize plants were improved by two folds upon biopriming with the fungus. Upon biopriming with *A. fumigatus* of the triploid maize at 400 mM NaCl, the shoot length and leaves areas were increased by about 1.7 folds, ensuring the positive physiological effect on the fungus on enhancing the physiological patterns of the salt-treated plants. Obviously, the biopriming of the non-salt treated maize with the fungus has no deleterious effect on the overall plant physiological features, as revealed from the various physiological parameters.

**Table 5:** Physiological parameters of the salt stressed triploid plant bioprimed with *A. fumigatus*. After 30 days of plant incubation, the different parameters were assessed

**Treatment**	Fresh Wt (g)	Dry Wt (g)	Leaf length	Leaf width	Shoot dry weight (g)	Number of fibrous roots	Root length	Shoot length	No. of leaves	Plant Wt (g)
C	6.0 ± 0.9** a**	1.1 ± 0.12** a**	74.3 ± 14.01** a**	2.49 ± 0.40** a**	2.5 ± 0.7** ab**	15.33 ± 1.52** a**	25.6 ± 5.03** a**	27 ± 4** a**	7.3 ± 0.5** a**	141.3 ± 48.4** a**
C + F	6.03 ± 0.10** a**	1.5 ± 0.09** b**	72.33 ± 3.05** a**	2.42 ± 0.37** a**	3.06 ± 0.11** a**	16.66 ± 1.15** a**	30.6 ± 2.08** a**	32.6 ± 2.51** a**	7.6 ± 0.57** a**	131.2 ± 21.2** a**
T4	2.6 ± 0.54 **b**	0.5 ± 0.15** c**	39.33 ± 4.04** b**	0.79 ± 0.15** b**	1.26 ± 0.25** c**	8.33 ± 1.52** b**	11.1 ± 1.0 **b**	13.33 ± 1.52** b**	5 ± 0** b**	23.6 ± 6.8** b**
T4 + F	3.7 ± 0.19** b**	0.8 ± 0.15 **bc**	47 ± 1** b**	0.95 ± 0.17** b**	2.06 ± 0.25** c**	7.33 ± 1.12** b**	17 ± 2.52** b**	17 ± 3.52** b**	6 ± 0** b**	33.69 ± 6.8** b**
**LSD 0.05**	**1.022**	**0.257**	**14.058**	**0.56**	**0.716**	**2.36**	**5.469**	**4.769**	**0.768**	**50.6**
***P***	0.0001 ***	0.0001 ***	0.0008 ***	0.0001 ***	0.0025 **	0.0000 ***	0.0002 ***	0.0000 ***	0.0001 ***	0.0009 ***

The mean values followed by different letters a, b, c with in the same column are significantly different (ONE Way ANOVA, Tukeyʾs test, *p* ≤ 0.05)

*n.s* non-significant, *LSD* the least significant difference

*Significant difference

**means highly significant

**Fig. 7:**
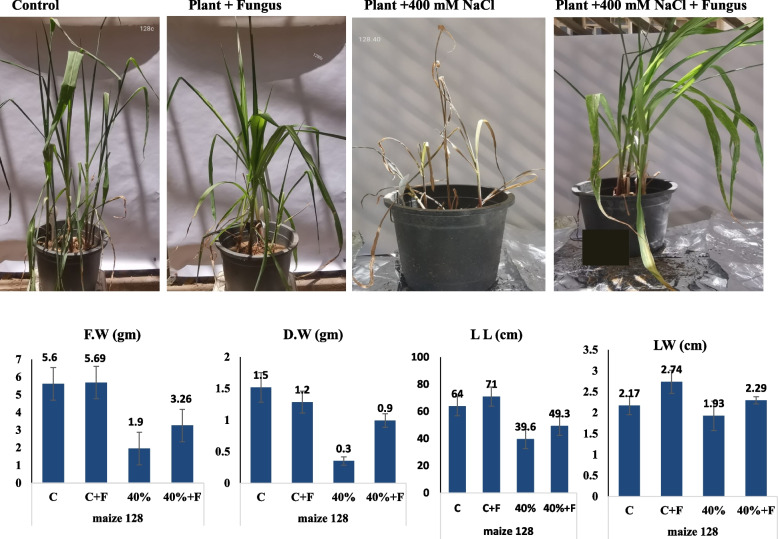
Morphological and physiological responses of the haploid triploid Maize (368) stressed with 400 mM NaCl and bioprimed with A. fumigatus. A, Visual growth of the salt stressed triploid Maize (368) bioprimed with A. fumigatus. B, Total Fresh weight (F.W), Dry Weight (D.W), Leave length (L.L) and Leave weight (L.W) of the seedlings after 30 days of growth

As well as, the effect of fungal biopriming of the haploid maize (128) on the resistance of the plant to salt stress was assessed (Fig. 7). From the general morphological features, the seedlings (128) at 400 mM NaCl were greatly improved upon fungal biopriming, compared to the non-primed plants. The seedlings viability was obviously improved counteracting the negative effect of salt upon fungal biopriming. The fresh weight, dry weight, leaves length and leaves widths of the plants were significantly increased by ~ two folds upon fungal biopriming compared to the salt-treated non-primed plants. At 400 mM NaCl, the plant fresh and dry weight was reduced from 5.5 g and 1.5 g, into 2.0 and 0.3 g, respectively. The different growth and physiological parameters of the salt stressed haploid maize bioprimed with *A. fumigatus* were summarized in Table 6.

**Table 6:** Physiological parameters of the salt stressed haploid plant bioprimed with *A. fumigatus*. After 30 days of plant incubation, the different physiological parameters were assessed

**Treatment**	Fresh Wt (g)	Dry Wt (g)	Leaf length	Leaf width	Shoot dry weight (g)	Number of fibrous roots	Root length	Shoot length	No. of leaves	Plant Wt (g)
C	5.6 ± 0.37 **a**	1.52 ± 0.5** a**	64 ± 3.6 **b**	2.17 ± 0.43** a**	2.26 ± 0.25** b**	17.6 ± 0.5** a**	29.66 ± 0.57** a**	27.66 ± 0.5** b**	9 ± 0.3 a	104.9 ± 26.1** ab**
C + F	5.69 ± 0.5** a**	1.28 ± 0.2** a**	71 ± 1 **a**	2.74 ± 0.67** a**	2.9 ± 0.36** a**	19 ± 1** a**	33.3 ± 3.055** a**	32.33 ± 2.0** a**	9 ± 0.4 b	146.2 ± 37.83** a**
T4	1.95 ± 0.1** b**	0.35 ± 0.2** b**	39.6 ± 0.5** c**	1.93 ± 1.10** a**	1.0 ± 0.15** c**	6.6 ± 0.57** b**	7.4 ± 0.34** c**	7.86 ± 0.15** d**	5 ± 0.7c	57.1 ± 31.67** b**
T4 + F	2.26 ± 0.2** b**	0.49 ± 0.1** b**	42.33 ± 0.5** c**	1.09 ± 0.06** a**	1.43 ± 0.05** c**	7.6 ± 0.57** b**	14.6 ± 0.5** b**	12.83 ± 0.2** c**	5 ± 0.6 b	34.8 ± 2.48** b**
**LSD 0.05**	**0.662**	**0.54**	**3.6**	**1.286**	**0.44**	**1.33**	**2.99**	**2.056**	**––-**	**52.63**
**P**	0.0000 ***	0.0026 **	0.0000 ***	**0.0955 ns**	0.0000 ***	0.0000 ***	0.0000 ***	0.0000 ***	**0.095 ns**	0.0051 **

The mean values followed by different letters a, b, c with in the same column are significantly different (ONE Way ANOVA, Tukeyʾs test, *p* ≤ 0.05)

*n.s* non-significant, *LSD* The least significant difference

*Significant difference

**means highly significant

**Fig. 8:**
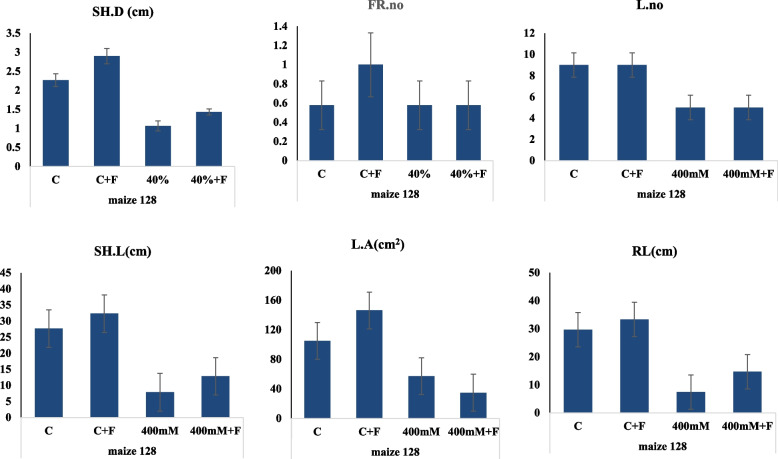
Physiological parameters of the salt stressed haploid plant bioprimed with A. fumigatus. After 30 days of plant incubation, the shoot density (S.D), fibrous roots number (FR, leaves number (L), Root length (RL), shoot length (SL), and leaves area of the triploid maize plants, were assessed

Biological biopriming of the haploid plant by *A. fumigatus* significantly increased stressed shoot fresh weight, and shoot density by 45% and 63% respectively. Meanwhile, in the inoculated stressed plants, the endophyte *A. fumigatus* significantly enhanced the root length and leaf area by 52% and 42% respectively, compared to the non-inoculated stressed plants (Fig. 8). The inoculation with the endophyte *A. fumigatus* did not change number of leaves and leaf area under control condition, in contrast, under salinity stress conditions, inoculated plants exhibited higher growth activity in shoot length by 27% than the non-inoculated stressed plants. Also, there is an improvement by 20% in root and shoot length parameters in the plants bioprimed with the fungus, compared to the non-inoculated under control conditions. When a plant faces a salt stress, it produces more fibrous roots in search for water, and according to the results, biological biopriming reduced the number of fibrous roots by 12%, confirming that the synergistic impact of fungal biopriming in stress reduction. The results discovered no significant change in these parameters when comparing inoculated control maize 368 to non-inoculated control maize 368.

The shoot fresh weight, shoot dry weight, leaf length, leaf width, shoot density and number of fibrous roots of maize plants subjected to salt stress decreased by half as compared to control plants, while biopriming maize grains with *A. fumigatus* significantly decreased the impact of the salt stress. The root length, shoot length, number of leaves and leaf area were significantly decreased by about 50% after exposing to salinity with 400 mM of NaCl.

### Physiological responses of the stressed plants bioprimed with the fungus

**Fig. 9:**
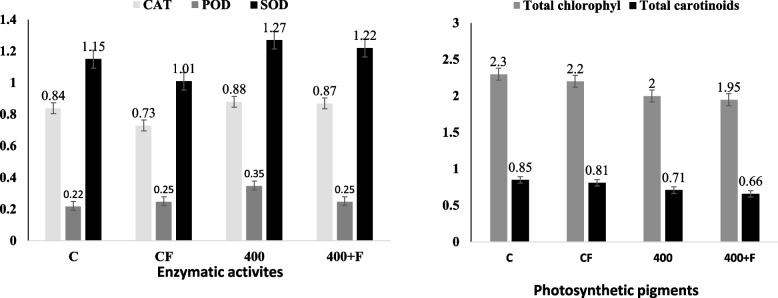
The antioxidant enzymatic activities and total pigments concentration of the triploid maize bioprimed with A. *fumigatus*

To assess the physiological response of the salt stressed plant in response to the fungal biopriming, the total antioxidant activities were assessed. The enzyme activities of the zero-salt plant, 400 mM NaCl, zero salt plant with fungus and 400 mM NaCl plant bioprimed with the fungus were determined. From the results, the total enzymes activities of the triploid maize plants were fluctuated with the salt concentration 400 mM, compared to zero salt (Fig. 9). At 400 mM salt, the activity of catalase in triploid maize (368) was increased by about 4.7%, compared to control, however, the catalase activity was decreased by 13% with the fungal treatment, compared to control, however, with the fungal biopriming of the stressed plants, catalase activity was increased by 1.13% in triploid plants 368 compared to the stressed plant (at 400 mM NaCl). The activity of peroxidase was determined on the control, salt stressed plant, and fungal bioprimed plants. From the results, the activity of peroxidase of the selected salt treatment 400 mM was significantly increased, compared to the control. The activity of peroxidase of the plant under salt stress (400 mM), compared to the non-stressed plant was significantly increased by 35.7%, in the triploid maize 368. The peroxidase activity of the stressed plant bioprimed with the fungus (C + F), compared to the non-stressed plants was reduced by 11.9%, in the triploid plants 368. The fungal treatment mitigates the salinity stress by decreasing the activity of POD by 22.8% in the triploid 368, compared to the stressed maize at 400 mM NaCl level. The activity of SOD was increased during salt treatment (400 mM) by 5.3%, compared to the control plant. In contrast, a significant decrease of SOD was observed under fungal treatment (C + F) by 1.52% in the triploid plants (368), compared to the control plant, however, the fungi biopriming on stressed plants decreased the SOD activity by 5% in 368 compared with 400 mM NaCl stressed Maize plants.

The total chlorophyll and carotenoids contents of the plant in response to the salt stress, bioprimed with the fungus were assessed (Fig. 9). The salt stress had a negative influence on the total chlorophyll and carotenoids contents, as revealed from the salt-stressed plants compared to the non-stressed plants (Control). At 400 mM of NaCl, the total chlorophyll of Maize 368 was slightly reduced to 2 mg/l, compared to the control. Similarly, a decrease of the total chlorophyll was observed in response to the fungal biopriming to 2.2 mg/l, compared with 2.2 mg/l in control plant, however, the fungal biopriming on stressed plants decreased the total chlorophyll activity to 1.95 mg/l compared with 2 mg/l in stressed plant with 400 mM. The results showed that salinity stress had a negative impact on total carotenoids contents. At 400 mM of NaCl, the total carotenoids of Maize 368 were slightly reduced to 0.71 mg/l, compared to 0.85 mg/l in control. Additionally, the total carotenoids were slightly decreased under fungal treatment compared to control plant, however, biopriming of the slat stressed plant with the fungus decreased the total carotenoids activity to 0.66 mg/l compared with 0.71 mg/l in stressed plant with 400 mM.

### RT-qPCR molecular expression analysis of the salt stress related genes in maize

**Fig. 10:**
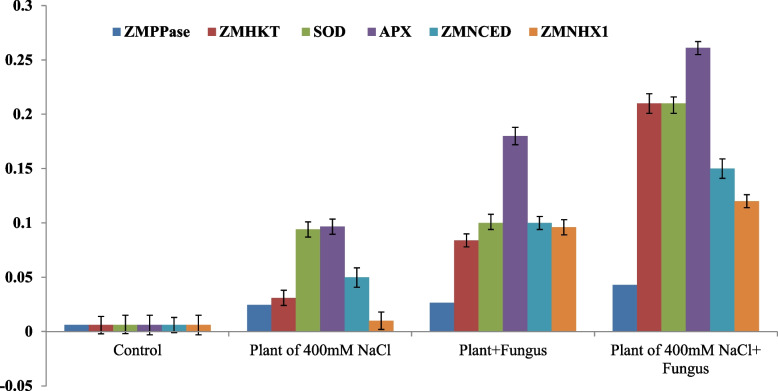
Molecular expression of the salinity stress-related genes of Maize in response to biopriming with A. fumigatus. The total RNA was extracted from the plants treated with zero salt, 400 mM NaCl and bioprimed with A. fumigatus, and the expressions of the genes ZMPPase, ZMKIT, SOD, APX, ZMNCED and ZMNHX1 were assessed by RT-qPCR, normalized to the actin A as house keeping genes

The molecular expression of the plant resistance to abiotic stress “salinity”; *sod4, apx2, ZmNHX1, ZmHKT1, ZmH*^+^*-PPase, ZmNCED* was assessed by the RT-qPCR, compared to *β-actin,* as house-keeping gene. The total RNA was extracted from the zero-salt plant, salt-stressed plants, and stressed plant bioprimed with the fungus, and reverse transcribed, and used as template for qPCR analysis. From the RT-qPCR analysis (Fig. 10), the expressions of the tested genes were strongly increased in response to salt stress, and with the fungal biopriming. The expression of *apx* gene was dramatically increased by about 2 and 3 folds, in response to fungal biopriming, compared to zero salt-stressed plants. The expressions of the *kit* and *sod* genes were increased by ~ 2 folds, compared to the zero salt plants, normalizing to the housekeeping gene. At 400 mM NaCl, the expression of the *ppase*, *kit, sod, apx,* and *nced* were obviously increased, compared to control, ensuring the implementation of these genes on the abiotic resistance of the plant. The higher expression of the tested genes with the fungal biopriming ensures the induction of these genes by the fungus to mitigate the toxicity of salt. With the plant biopriming with *A. fumigatus,* the expression of *ppase* and *apx* genes were up-regulated by ~ 2 and 3 folds, compared to the non-inoculated plant. Also, the expression of *ZMNHX1* gene was up-regulated by twofold, in response to the fungal biopriming. These results indicate that zea maize plant experienced less stress induced by salinity in presence of *A*. *fumigatus* which reflect the positive effect of biopriming in reducing salt stress in Zea maize.

## Discussion

Zea mays is one of the most widely grown crop cultivated in nearly 150 M ha area across the globe [51, 52]. Maize is one of the moderately sensitive crops to salt stress, so, salinity is one of the most serious threats to the plant growth and productivity worldwide [53]. Salinity has a detrimental effect on the plant growth for its osmotic effect, nutritional imbalance, ion toxicity, oxidative stress, lowering the membrane stability and plant performance deterioration [14]. Thus, molecular and metabolic manipulation to increase the salt resistance of maize is the main challenge. Among the common approaches to mitigate the salt stress and increase the plant resistance to abiotic stress, is the seed priming with their indigenous endophytic microorganisms [54]. Endophytic microorganisms colonize the xylem and intercellular spaces of parenchyma tissue [55, 56]. The main physiological benefits of the endophytic microorganisms to their plant host is modulating the intrinsic plant defense towards the natural biotic invaders via production of antibiotics, siderophores, growth hormones, bacteriocins, and antimicrobial peptides [57]. Microbes inhabiting seeds are receiving more attention due to their crucial roles in the process of seed germination and seedling growth [58, 59], by solubilizing the phosphate and potassium, producing growth hormones such as auxin and cytokinin [60]. The seeds endophytes provide the tolerance to the plants from different biotic and abiotic stresses, thereby improving their overall fitness [61, 62]. So, the objective of this work was to increase the salt resistance of maize via biopriming with their fungal endophytes. The haploid (128) and triploid (368) seeds of maize were selected as model verities for assessing their resistance to salt stress. The salt tolerance of the haploid and triploid maize was assessed by irrigating the plant seedlings with different NaCl (100–500 mM) then observing the plant viability. The overall morphological appearance, growth and viability of the seedlings were drastically affected by the high salt concentration in a salt concentration- dependent manner. At 500 mM NaCl, the fresh weight, shoot length and leaves area of the triploid maize were reduced by about 5, 9.6 and 3.4 folds, respectively, compared to zero salt plant. From the comparative physiological parameters, the seedlings of triploid maize were slightly resistance to the salt stress than haploid one, as revealed from the fresh weight, shoot length and leaves area of the plants. Similar results ensure the negative effect of high NaCl concentration on the soybean and rice, by inhibiting the development and growth of shoot and root systems of the plants [63–65].

The frequency and identity of the endophytic fungi inhabiting the plants at different salt concentration were determined. An obvious fluctuation on the incidence, identity of the fungal endophytes was observed from the zero-salt to salt-stressed maize plants. Among the conspicuous endophytic fungi, the isolate *Aspergillus fumigatus* EFBL-My was obviously emerged with at 400 and 500 mM. The isolate has been identified based on their morphological features [36, 50], and confirmed based on their ITS sequence, as deposited on genbank with accession # PQ200673. Similar paradigm of the endophytes for *Triticum aestivum* was reported*,* with an obvious fluctuation with salt stress [21]. To assess the plant potency to alleviate the salt stress in response to fungal-biopriming, the haploid and triploid maize seeds were bioprimed with *A. fumigatus*, grow at salt stress (400 mM), and the morphological growth and various physiological parameters were assessed. Unlike to the higher viability of the fungal bioprimed seedlings, the viability of the non bioprimed plant seedlings was strongly affected by 400 mM NaCl. The various physiological parameters of the triploid and haploid maize seedlings bioprimed with *A. fumigatus* were significantly improved, compared to the non-bioprimed plants. Also, the shoot density, fibrous roots, leaves number, root length, shoot length, and leaves areas of the stressed-plants, were significantly improved upon biopriming with *A. fumigatus*. Upon *A. fumigatus* biopriming, the shoot length and leaves areas of triploid maize at 400 mM NaCl, were increased by ~ 1.7 folds, ensuring the positive physiological effect by the fungus on enhancing the different salt resistance machineries. As well as, growth viability, fresh weight, dry weight, leaves length and leaves widths of the haploid maize seedlings were significantly increased by about two folds upon *A. fumigatus* biopriming, compared to the salt-treated non-primed plants. Biological biopriming of the haploid plant by *A. fumigatus* significantly increased the stressed shoot fresh weight, and shoot density by 45% and 63% respectively. Similar results were reported for Wheat bioprimed with *Phanerochaete* [21]*.* Biopriming of wheat grains with *P. chrysosporium* significantly alleviates the salt stress and increases the growth parameters, photosynthetic pigments, osmolytes, alleviating the overall oxidative damage [21, 66].

The higher growth rate and developments of plants with the fungal bioprimed seeds, might be attributed to the enhanced nutrient use efficiency and cell cycle regulation and cell elongation processes [67]. The fading and decreasing the viability of the stressed-plants a might be due to the decrease in chlorophyll contents, that being consistent with those reported for rice [68], fenugreek [69], and sorghum [70], by suppression of chlorophyll biosynthesis enzymes like Rubisco and PEP carboxylase [71], and the activation of chlorophyllase, a chlorophyll-degrading enzyme by cleaving the phytol tail of chlorophyll [72]. Consistently, biopriming of maize with *Trichoderma atroviride* before NaCl expose, increase the cell viability by increasing the biosynthesis of chlorophyll and carotenoid contents in maize seedlings [73]. *Trichoderma citrinoviride* bioprimed salt-stressed maize resulted in a slight increase on chlorophyll and carotenoids contents, compared to un-primed plants [74]. Similarly, the improved tolerance of plant upon fungal biopriming could be via modulating the photosynthesis II and antioxidant activities [75]. Biopriming of carrot and wheat plants with *Paecilomyces lilacinus* and *Trichoderma* spp increases the chlorophyll contents and improves the photosynthetic pigments (Nesha and Siddiqui, 2017), that could be attributed to the synthesis of phytohormones, namely axins, gibberellins and cytokinins in the plants (Martínez-Medina et al., 2014). The total antioxidant enzymatic activities of the seedlings of maize were fluctuated with the salt concentration 400 mM, compared to zero salt. At 400 mM salt, the activity of catalase, peroxidase, and SOD in triploid maize was increased by about 5 folds compared to the control. Physiologically, the higher antioxidant activity is usually correlating with the down-regulating of the reactive oxygen species (ROS). Consequently, the ROSs can cause oxidative destruction of the cellular macromolecules DNA, proteins and lipids [76]. So, the endogenous defensive mechanism that controls ROS levels in the plant cells is developed by complex enzymatic (superoxide dismutase (SOD), catalase, ascorbate peroxidase, glutathione reductase, glutathione peroxidase, glutathione S-transferase) and non-enzymatic compounds (ascorbic acid), glutathione, phenolic acids, alkaloids, carotenoids, flavonoids, and α-tocopherol [77–79]. These mechanisms collectively regulate the redox state of plant cells under stress [80]. The antioxidants enzymes “catalase, peroxidase, and superoxide dismutase” are essential in scavenging the reactive oxygen species (ROS) [81]. The salt stress had a negative influence on total chlorophyll and carotenoids contents, compared to the non-stressed plants. Similarly, the salt stress caused a decrease in chlorophyll content in rice [68], and sorghum [70] by suppressing the chlorophyll biosynthesis enzymes like Rubisco and PEP carboxylase [71] and the activation of chlorophyllase by cleaving the chlorophyll phytol tail [72]. Consistently, *Trichoderma atroviride* seed biopriming, the chlorophyll and carotenoid contents were increased in maize seedlings [73, 82]. Consistently, the *Ascophyllum nodosum* seaweed biomass enhances the tomato plant grown under salinity stress, as revealed from chlorophyll contents, antioxidant enzymatic activities [83]. Biopriming of barley with *A. ochraceus* strongly improve the plant resistance to salt stress, as revealed from the plant morphological traits and antioxidant enzymatic activities, ensuring the role of fungus for production of indole acetic acid [84].

To validate the physiological parameters of the stressed plant in response to biopriming, the molecular expression of selected abiotic stress- resistance genes “*sod4, apx2, nhx1, hkt1*, *H* + *-ppase,* and *nced*” was assessed by RT-qPCR. The expressions of the tested genes were strongly increased in response to salt stress, and with the fungal biopriming. The expression of *hkt*, *apx,* and *nced* genes was dramatically increased by about 3 folds, in response to fungal biopriming, compared to zero salt-stressed plants. The molecular expression of these genes ensures their implementation on salt tolerance, in response to fungal-plant biopriming. The higher expression of the tested genes by the fungal biopriming over the control ensures the induction of these genes by the fungus to mitigate the toxicity of salt. From the molecular expression analysis, the expression of the tested genes was closely matched with the measured physiological parameters. The higher expression of the H^+^-ppase (encoding H + -pumping pyrophosphatase), hkt1 (encoding high-affinity K + transporter 1), and nhx1 (encoding Na + /H + antiporter) mediates processes of Na + isolation, export, and recirculation were increased in the salt-stressed maize in response to fungal biopriming, as consistent with those reported by previous studies [85]. The expression of NCED gene (encoding 9-cis-epoxycarotenoid dioxygenase), a key gene in ABA production, has been significantly increased in salt-stressed plant seedlings bioprimed with *A. fumigatus,* the ABA is a crucial plant hormone that regulates its resistance to abiotic stress [86, 87] via modulating the ion channel performance and closure of stomatal pores in plant guard [88–90]. The SOD and peroxidase, transforming superoxide into H_2_O_2_ followed by oxygen release, has been reported to be highly increased in slat-stressed plants bioprimed with *A. fumigatus* [91]. Plant cells may be able to reduce the toxicity of Na^+^ in salty environments by a number of strategies, such as limiting the intake of Na^+^, recycling Na^+^ through the xylem streams to the root system, isolating Na^+^ in vacuoles, and exporting it from the cell [92]. The role of plant antioxidant machinery in reducing the stresses like salinity has also been reported [93, 94].

In conclusion, abiotic stress particularly, soil salinity, is one of the most crucial factors affecting maize growth and productivity. Biopriming of the salt stressed haploid and triploid seeds of maize seeds with *A. fumigatus* greatly improve the plant viability, and growth as revealed from the morphological appearance, different physiological parameters of the seedlings, antioxidant activities, in addition to the molecular expression of the salt-resistance related genes. As well as, seed bio-priming of maize with the salt-tolerant endophytic *A. fumigatus* modulates the biochemical responses and provides ecological fitness to the maize (*Zea mays* L.) plants grown in saline soil.

## Data Availability

The datasets of the current study are available from the corresponding author on a reasonable request.
